# Impact of Pregnancy on Weight Loss After Endoscopic Sleeve Gastroplasty

**DOI:** 10.1007/s11695-023-06745-6

**Published:** 2023-08-05

**Authors:** Giorgio Carlino, Ariel A. Benson, Vincenzo Bove, Valerio Pontecorvi, Martina De Siena, Maria Valeria Matteo, Annarita Farina, Giulia Polidori, Laila Vinti, Giulia Giannetti, Guido Costamagna, Cristiano Spada, Ivo Boškoski

**Affiliations:** 1 Digestive Endoscopy Unit, Fondazione Policlinico Universitario Agostino Gemelli IRCCS, 00168 Rome, Italy; 2 Center for Endoscopic Research Therapeutics and Training (CERTT), Università Cattolica del Sacro Cuore, Rome, Italy; 3 Institute of Gastroenterology and Liver Diseases, Department of Internal Medicine, Hadassah Medical Center and Faculty of Medicine, Hebrew University of Jerusalem, Jerusalem, Israel

**Keywords:** Obesity, Pregnancy, Bariatric endoscopy

## Abstract

**Purpose:**

Obesity and pregnancy are strictly related: on the one hand, obesity—one of the most common comorbidities in women of reproductive age—contributes to infertility and obesity-related pregnancy complications, whereas pregnancy is a condition in which, physiologically, the pregnant woman undergoes weight gain. Endoscopic sleeve gastroplasty (ESG) may be used for the treatment of obesity in women of childbearing age.

**Materials and Methods:**

A retrospective analysis was conducted to evaluate weight trajectories, the evolution of obesity-related comorbidities, and lifestyle modification in women who became pregnant after ESG. A comparison was made between childbearing-age women who became pregnant after ESG and non-pregnant women.

**Results:**

A total of 150 childbearing-age women underwent ESG at a large tertiary medical center. Of these, 11 patients (33.4 ± 6.2 years) became pregnant after the procedure, following a mean time interval of 5.5 ± 3.9 months. Three women (two affected by polycystic ovary syndrome) reported difficulty getting pregnant before undergoing ESG. The mean preconception BMI was 31.9±4.0 kg/m^2^ (−7.24 ± 4.0 kg/m^2^ after ESG). Total body weight loss (TBWL, %) was 18.08 ± 8.00, 11.00 ± 11.08, and 12.08 ± 8.49, at the beginning of pregnancy, at the delivery, and at the first follow-up (19.6 ± 7.8 months after ESG). TBWL of at least 5% was achieved before pregnancy in all patients (73% reached a TBWL ≥ 10%).

No significant differences in weight loss and QoL were found between the pregnancy and non-pregnancy groups up to 24 months after ESG.

**Conclusions:**

Endoscopic sleeve gastroplasty allows for adequate weight loss before and after pregnancy in patients with obesity.

**Graphical Abstract:**

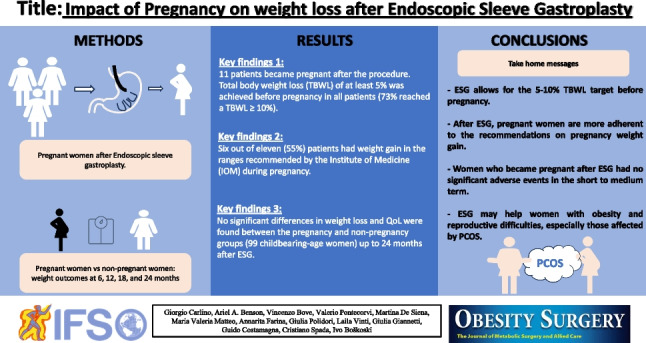

## Introduction

Obesity, defined by the World Health Organization as a body mass index (BMI) ≥ 30 kg/m^2^, is a chronic, relapsing, and multifactorial disease that has become a global concern. By the year 2030, it is predicted that 1 in 5 women and 1 in 7 men will live with obesity, equating to over 1 billion people globally [1].

Obesity is the most common health problem in women of reproductive age [2], with 39.7% of women 20 to 39 years of age being affected. In women wishing to become pregnant, obesity represents an obstacle to conception [3]. Furthermore, being obese at the beginning of pregnancy not only represents a risk factor for both gestational and postpartum maternal complications, but also it can adversely affect fetal, neonatal, and infant outcomes [4]. For this reason, the *American College of Obstetricians and Gynecologists* (ACOG) recommends encouraging optimal control of obesity, regardless of the therapeutic strategy, before pregnancy [4]. Even a small weight reduction before pregnancy may be associated with improved pregnancy outcomes because medications for weight management are not recommended during pregnancy. However, in the USA, among women with a live birth in 2020, more than half began pregnancy being overweight (26.7%) or obese (29.5%) [3]. In addition, ACOG recommends recording pre-pregnancy BMI at the initial prenatal visit, which should be used to provide diet and exercise counselling guided by the Institute of Medicine (IOM) recommendations for gestational weight gain during pregnancy. However, gestational weight gain was within the recommended range for only 32% of women giving birth to full-term singleton infants in 2015, with 48% gaining more weight [5].

For women in childbearing age with overweight or obesity, weight loss of 5 to 10% could be a possible target since it has been shown to improve metabolic conditioning before pregnancy, increase the likelihood of conception, and reduce the risk of preeclampsia [3]. Unfortunately, lifestyle interventions often fail when people revert to previous eating and exercise habits. Bariatric surgery is a more invasive but more effective option for weight loss in the long term [6]. Most patients who undergo bariatric surgery are women, especially those of childbearing age [7]. Furthermore, in a large meta-analysis, patients who underwent bariatric surgery before pregnancy had lower rates of several adverse obstetric outcomes when compared with controls who were matched for pre-surgery BMI [8]. However, the bariatric surgery patients showed an increase in small-for-gestational-age infants, intrauterine growth, and preterm deliveries as compared with controls [8], resulting in an unclear risk-benefit ratio.

At the same time, pregnancy is associated with physiological and anatomical changes because of hormonal variations that could potentially affect surgical weight loss outcomes. Currently, only a few retrospective studies have evaluated the effects of pregnancy on the trajectories of weight loss and, therefore, on the effectiveness of bariatric surgery, showing that post-operative weight loss did not diminish in the long term [9]–[11].

Despite the obvious benefits, about 1% of the candidates undergo bariatric surgery [12], with an evident unmet clinical need. To try to fill this gap several techniques of bariatric endoscopy, minimally invasive, scar-free, and potentially more accepted by patients have made their entry into clinical practice. Among them, endoscopic sleeve gastroplasty (ESG) is an emerging minimally invasive, organ-sparing, endoluminal bariatric procedure that can reduce gastric volume and modify peristalsis by the placement of full-thickness sutures. More effective than dietary treatment [13] and potentially not inferior to the surgical counterpart the sleeve gastrectomy [14], ESG is spreading more and more. Additionally, a systematic review with meta-analysis (involving a total of 1772 patients) proved the efficacy and the durability of the procedure with mean total body weight loss (TBWL) of 15.1%, 16.5%, and 17.2% respectively at 6 months, 12 months, and 18–24 months with a low post-ESG rate of severe adverse events (2.2%) [15].

On the one hand, the promising results of intragastric balloon treatment [16], albeit retrospective, certainly make the idea of exploring the effects and effectiveness of ESG in the context of obesity-related infertility appealing. On the other hand, since these patients are intrinsically interested in weight, understanding the effects of pregnancy on post-operative weight loss in bariatric endoscopy is essential to guide doctors in counselling patients in this field.

However, due to the relatively recent genesis, no study has yet evaluated the effects of ESG on pregnancy in women with obesity, both in terms of preconception weight reduction and gestational outcomes nor the possible effects of gestation on weight-loss outcomes.

In this study, we aimed to investigate the impact of pregnancy on ESG outcomes and the possible effects of ESG as a preconceived therapy in women with obesity who got pregnant after endoscopic surgery.

## Methods

### Study Design, Ethics, and Participants

A retrospective analysis was performed on a prospective dataset collecting data on all patients with obesity treated with ESG to evaluate the impact of pregnancy on weight loss after ESG at an Italian tertiary medical center. All patients underwent ESG after approval by the local multidisciplinary team, including surgeons, endoscopists, endocrinologists, nutritionists, and psychologists.

All childbearing-age (between 18 and 45) subjects who underwent ESG were included in the analysis. All pregnant patients after ESG were identified. Pregnancy status was defined as having a written diagnosis in the medical chart.

The weight trajectories at 6, 12, 18, and 24 months after ESG of the identified pregnancy patient were then compared to that of women of childbearing age (between 18 and 45) who underwent ESG and who did not get pregnant after the procedure. Childbearing-age patients who were lost to follow-up/have not reached the follow-up before 6 months were excluded from the analysis.

The institutional ethical committee approved this clinical investigation (register no. 19201/18, ID 2083). Informed consent was obtained from all individual participants included in the study. The study was conducted according to good clinical practice guidelines and adhered to the Declaration of Helsinki.

### Data Collection

For each pregnant patient, the following pre-conceptional parameters were evaluated: possible difficulties in getting pregnant before the ESG (defined as unsuccessful attempts for more than 12 months reported by the patient), being affected by polycystic ovary syndrome (PCOS), whether the patient was primiparous or not, the preconception BMI, and the time (months) elapsed between the ESG and pregnancy.

For the non-pregnant cohort, the following pre-conceptional parameters were evaluated: possible difficulties in getting pregnant before the ESG and being affected by polycystic ovary syndrome (PCOS).

**Fig. 1:**
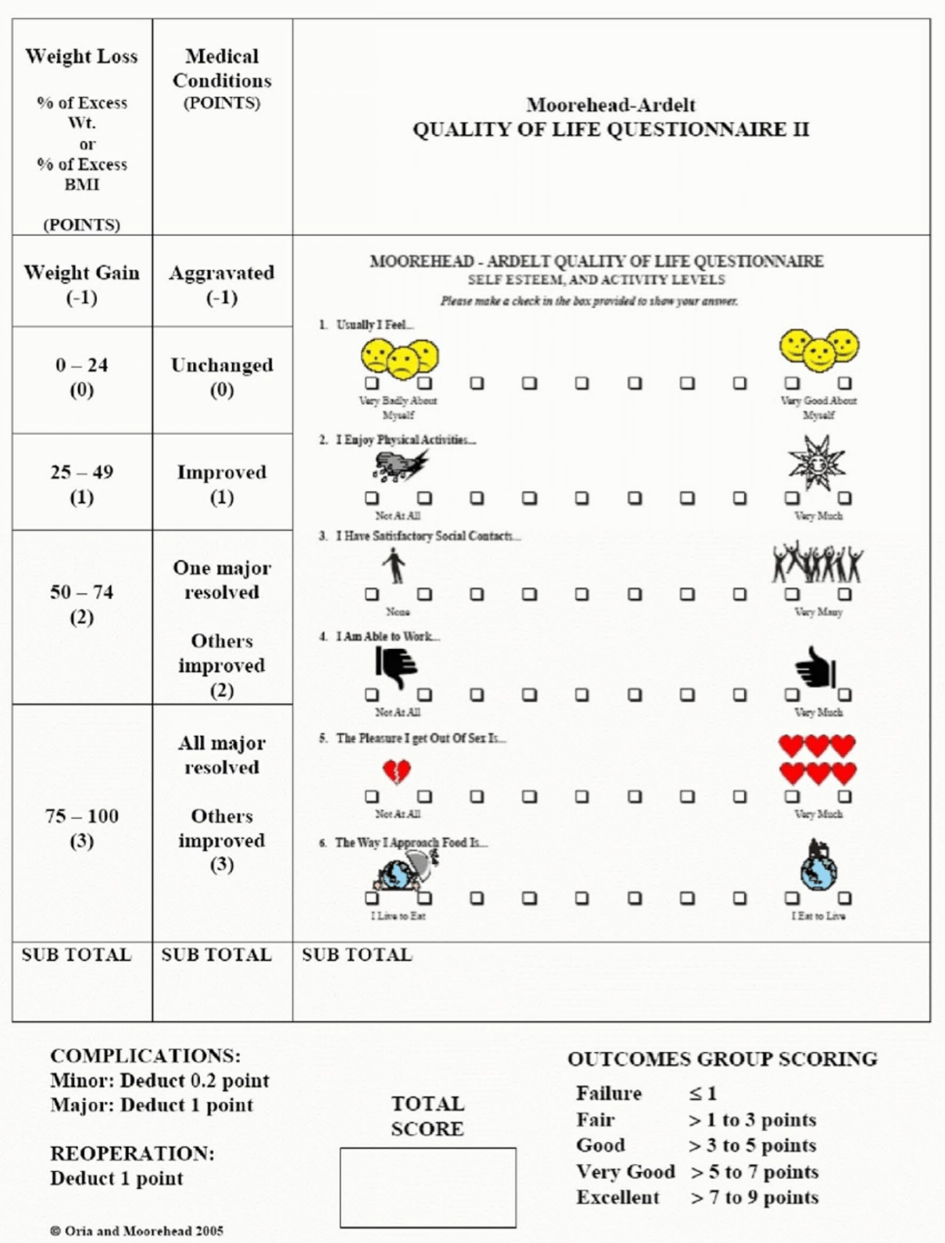
Bariatric Analysis and Reporting Outcome System BAROS (Moorehead-Ardelt quality of life questionnaire—this instrument is copyright protected, and licensing for publication in this paper was obtained from Dr. Melodie Kay Moorehead at drmoorehead.com)

Weight loss indices (weight loss (WL), total body weight loss (TBWL), excess weight loss (EWL), BMI, BMI loss (BMIL)), weight gain during pregnancy, the evolution of obesity-related comorbidities, the Bariatric Analysis and Reporting Outcomes System questionnaire (BAROS, Fig. 1), and quality of life (QoL, based on the Moorehead-Ardelt quality of life questionnaire II, Fig. 1) were assessed at the beginning and end of pregnancy and the first postpartum visit. Specifically, the Moorehead-Ardelt quality of life questionnaire II measures the patient’s subjective impression of QoL through the specific item for each of the following areas: (1) *general self-esteem*, (2) *physical activity*, (3) *social contacts*, (4) *satisfaction concerning work*, (5) *pleasure related to sexuality*, and (6) *eating behaviors*. Each item is weighted on a 10-point Likert scale (from −0.5 to +0.5) [17].

All weight loss indices were calculated as recommended by the American Society for Metabolic and Bariatric Surgery (ASMBS) [18].

### Procedure

ESG was performed under general anesthesia with the Overstitch™ (Apollo Endosurgery, Austin, TX, USA) suturing system mounted on a double channel therapeutic endoscope (2TGIF -160/2TGIF -180; Olympus Medical Systems Corp., Tokyo, Japan) or the Overstitch SX™ (Apollo Endosurgery, Austin, TX, USA) suturing system mounted on a single-channel endoscope (GIF-H190/GIF-HQ190; Olympus Medical Systems Corp., Tokyo, Japan).

Using the suturing device, 2/0 polypropylene running sutures were placed, beginning from the level of the incisura angularis to 2–3 cm below the gastroesophageal junction. Usually, each suture started at the anterior wall of the sleeve, with subsequent bites progressing along the greater curvature and to the more proximal posterior wall. At our center, the average duration of the procedure is around 45 min.

### Statistical Analysis

Categorical variables were described by their frequencies. Continuous variables were described by their means ± standard deviations and/or their median (interquartile range).

The Mann-Whitney *U* tests were used to compare pregnant and not pregnant women with a 0.05, 2-sided significance level.

## Results

Between January 2018 and December 2021, two hundred and seventy-nine women underwent ESG. One hundred and twenty-nine women (46.2%) were older than 45 years old.

Out of a total of 150 patients of childbearing age, 18 patients (12.0%) reported a desire for pregnancy associated with unsuccessful attempts in the 12 months preceding ESG.

Of the one hundred and fifty childbearing-age women, 110 (73.0%) met the inclusion criteria, while 17 subjects were lost to follow-up and 23 did not reach the 6-month follow-up.

Out of the total, 11 patients (11/110 (10.0%), mean age 33.4 ± 6.2 years) became pregnant after ESG. Among the 23 patients excluded because they have not yet reached the follow-up visit at 6 months, none remained pregnant after ESG.

### Pre-conceptional Parameters

Among the pregnant women, three of them reported difficulty in becoming pregnant before the ESG: two were affected by PCOS. An additional patient had a history of PCOS but did not report difficulty in becoming pregnant. Six patients (55%) were primiparous, two of them with a history of PCOS.

Mean preconception BMI was 31.9 ± 4.0 kg/m^2^ (with a mean reduction of 7.24 ± 4.0 kg/m^2^ after ESG), with seven subjects affected by obesity (4 were Class I (BMI 30–35 kg/m^2^) and three Class II obesity (BMI 35–40 kg/m^2^)) and four subjects with overweight (BMI > 25 kg/m^2^). The mean interval between the ESG and pregnancy was 5.5 ± 3.9 months, with nine subjects becoming pregnant within 12 months after the procedure. No miscarriages were reported.

### Weight Trajectories, the Evolution of Comorbidities, BAROS Questionnaire, and QoL

At the beginning of pregnancy, mean WL, TBWL, EWL, and BMIL were 19.9 ± 10.21 kg, 18.08 ± 8.00%, 52.26 ± 20.96%, and 7.24 ± 3.99 kg/m^2^, respectively. At the beginning of pregnancy, all the subjects reached a TBWL ≥ 5%, with eight women (73%) reaching a TBWL ≥ 10%. Whereas, at the delivery (14.5 ± 4.2 months after ESG), mean WL, TBWL, EWL, and BMIL were 12.18 ± 12.93 kg, 11.00 ± 11.08%, 31.35 ± 34.19%, and 4.37 ± 4.20 kg/m^2^, respectively, with one patient experiencing a complete weight regain.

At the first follow-up visit after the delivery (mean interval, 5.18 ± 3.6 months), one patient underwent a second ESG procedure (Re-ESG) for weight regain. For the other ten subjects, mean WL, TBWL, EWL, and BMIL were 12.90 ± 9.95 kg, 12.08 ± 8.49%, 35.91 ± 26.64%, and 4.70 ± 3.23 kg/m^2^, respectively. Two subjects had a TBWL< 5%, eight women reached a TBWL ≥ 5%, with five women reaching a TBWL ≥ 10%.

Among the 10 pregnant patients who underwent prior ESG, seven patients reached the last follow-up visit (29.6 ± 13.9 months after ESG) after 16.7 ± 12.2 months from the delivery. At the last follow-up visit, mean WL, TBWL, EWL, and BMIL were 19.33 ± 11.84 kg, 17.42 ± 9.99%, 50.19 ± 32.05%, and 6.99 ± 4.07 kg/m^2^, respectively. One subject had a TBWL< 5%, six women reached a TBWL ≥ 5%, with five reaching a TBWL ≥ 10%. Also noteworthy is the fact that one patient started her second pregnancy 24 months after ESG with a 19.85% TBWL at the beginning of pregnancy. No adverse events were recorded in the perioperative period.

Three patients were affected by hyperinsulinemia (H-INS), while an additional patient had both H-INS and high blood pressure (HBP). The patient with H-INS and HBP underwent resolution of her comorbidities both before and during pregnancy. One of the three patients with H-INS had an improvement during gestation, while the other two had no changes in their condition.

The mean BAROS (QoL) was 5.57 ± 1.72 (2.57 ± 1.72) at the beginning of the pregnancy, 3.30 ± 1.62 (2.11 ± 0.67) at the delivery, 3.53 ± 1.98 (2.16 ± 1.08) at the first follow-up visit, and 2.50 ± 2.50 (1.50 ± 1.50) at the last follow-up visit.

Six out of 11 patients (54.5%) had pregnancy weight gain in the range recommended by IOM. Specifically, 77% and 25% of the women with obesity and overweight at the beginning of pregnancy, respectively, gained weight within the recommendation.

The weight parameters are summarized in Table 1.

**Table 1:** Weight outcomes of the eleven pregnant patients after endoscopic sleeve gastroplasty in Rome (10 patients) and Israel (1 patient). Data are reported as mean value ± standard deviation

Population	WL (kg)	EWL (%)	TBWL (%)	BMI (kg/m^2^)	Δ BMI (kg/m^2^)	EBMIL (%)	BAROS	QoL
Beginning of pregnancy^1^ (*N* = 11)	19.09 ± 10.21	52.26 ± 20.96	18.08 ± 8.00	31.86 ± 4.02	7.24 ± 3.99	52.26 ± 20.96	5.57 ± 1.72	2.57 ± 0.53
End of pregnancy^2^ (*N* = 11)	12.18 ± 12.93	31.35 ± 34.19	11.00 ± 11.08	34.73 ± 6.03	4.37 ± 4.20	31.35 ± 34.19	3.30 ± 1.62	2.11 ± 0.67
First follow-up after delivery^3^ (*N* = 10*)	12.90 ± 9.95	35.91 ± 26.64	12.08 ± 8.49	34.61 ± 6.39	4.70 ± 3.23	35.91 ± 26.64	3.53 ± 1.98	2.16 ± 1.08
Last follow-up after delivery^4^ (*N* = 7)	19.33 ± 11.84	50.19 ± 32.05	17.42 ± 9.99	33.83 ± 7.51	6.99 ± 4.07	50.19 ± 32.05	2.50 ± 2.50	1.50 ± 1.50

*WL* absolute weight loss, *EWL* excess weight loss, *TBWL* total body weight loss, *BMI* body mass index, *EBMIL* excess body mass index loss, *BAROS* Bariatric Analysis and Reporting Outcome System Questionnaire; *QOL* quality of life based on the Moorehead-Ardelt quality of life questionnaire II. ^1^Mean interval of 5.5 ± 3.9 months from ESG; ^2^Mean interval of 14.5 ± 4.3 months from ESG; ^3^Mean interval of 19.6 ± 7.8 months from ESG; ^4^Mean interval of 29.6 ± 13.9 months from ESG. *One patient underwent re-ESG

### Comparison Between Pregnant and Not Pregnant Women

For the 11 pregnant women (PW), the follow-up rate was 100% at 6 and 12 months, 81% (9/11) at 18 months, and 64% (7/11) at 24 months. Whereas for the 99 childbearing-age women who did not become pregnant after ESG (NPW), the follow-up rate was 100%, 81%, 49.5%, and 33.3%, at 6, 12, 18, and 24 months, respectively.

Age baseline weight, BMI, ideal weight, and excess weight were similar in both groups (Table 2). No significant differences were found in WL, TBWL, EWL, BMI, BAROS, and QoL between PW and NPW up to 24 months after ESG, as shown in Table 3.

**Table 2:** Baseline comparison between women who got pregnant after endoscopic sleeve gastroplasty (ESG) and fertile women who did not get pregnant after ESG

Population	Age (year)	Weight (kg)	BMI (kg/m^2^)	Ideal weight (kg)	Excess weight (kg)
PW (*N* = 11)	35.0 (9.0)	99.0 (16.5)	37.0 (4.3)	65.6 (4.4)	33.2 (10.1)
NPW (*N* = 99)	37.0 (11.0)	100.0 (16.0)	36.7 (5.1)	66.4 (7.8)	32.4 (15.3)
*p*	*0.2901*	*0.6147*	*0.441*	*0.5478*	*0.4977*

*PW* pregnant women; *NPW* non-pregnant women, *BMI* body mass index, *N* number of subjects

**Table 3:** Comparison between pregnancy group (PW) and non-pregnancy group (NPW) after 6, 12, 18, and 24 months from endoscopic sleeve gastroplasty. Data are reported as median (interquartile range). The Mann-Whitney *U* test was used to compare pregnant and not pregnant women with a 0.05 2-sided significance level

	6 months		*p*	12 months		*p*	18 months		*p*	24 months		*p*
	PW	NPW		PW	NPW		PW	NPW		PW	NPW	
Numerosity	11	99		11	81		9	49		7	33	
WL (kg)	20.0 (15.0)	18.0 (9.0)	*0.51*	23.0 (21.5)	15.0 (14.0)	*0.77*	12.0 (10.0)	13.0 (18.0)	*0.78*	10.0 (18.5)	13.0 (16.0)	*0.84*
TBWL (%)	20.6 (11.0)	17.4 (8.7)	*0.52*	20.2 (18.0)	15.9 (12.7)	*0.94*	12.2 (9.2)	12.5 (17.1)	*0.50*	10.1 (16.1)	14.9 (16.1)	*0.66*
EWL (%)	63.7 (26.0)	54.9 (31.4)	*0.67*	44.7 (48.5)	51.8 (39.4)	*0.66*	30.1 (28.4)	48.0 (52.7)	*0.38*	31.5 (42.1)	50.7 (50.4)	*0.66*
BMI (kg/m^2^)	30.5 (6.7)	30.4 (4.5)	*1*	32.2 (5.2)	30.9 (5.8)	*0.55*	35.7 (6.1)	30.9 (6.9)	*0.18*	37.0 (9.4)	31.8 (8.7)	*0.43*
BAROS	4.3 (1.4)	4.0 (1.8)	*0.26*	4.8 (2.6)	4.0 (2.6)	*0.37*	3.0 (1.5)	2.8 (4.3)	*0.52*	2.5 (2.8)	3.0 (4.4)	*0.72*
QoL	2.5 (1.1)	2.0 (1.0)	*0.12*	2.5 (1.0)	2.0 (1.3)	*0.07*	2.0 (1.0)	1.4 (1.9)	***0.05***	2.0 (0.8)	1.5 (2.0)	*0.17*

The bolded value is the only one that is statistically significant

*PW* pregnant women, *NPW* non-pregnant women, *WL* weight loss, *TBWL* total body weight loss, *EWL* excess weight loss, *BMI* body mass index, *BAROS* Bariatric Analysis and Reporting Outcome System questionnaire; *QoL* quality of life based on the Moorehead-Ardelt quality of life questionnaire II

## Discussion

Pregnancy means weight gain for definition, and it is strictly related to obesity, which is the most common health comorbidity in women of reproductive age [2]. In women wishing to become pregnant, obesity represents an obstacle to conception, associated with menstrual dysfunction, a lower likelihood of conception per cycle, subfertility or infertility, and miscarriage [3]. In this regard, obesity is thought to have multiple actions on the hypothalamic-pituitary-ovarian axis. In particular, the increased circulating levels of insulin, related to obesity, seem to stimulate ovarian production of androgens, which are in turn aromatized to estrogen in adipose tissue. The negative feedback on the hypothalamic axis imposed by elevated estrogen levels causes menstrual abnormalities and ovulatory dysfunction evident in women with obesity and especially in those with polycystic ovarian syndrome (PCOS) [19, 20].

Furthermore, obesity is a risk factor for both gestational and postpartum complications, and it can adversely impact fetal, neonatal, and infant outcomes [4]. For this reason, the *American College of Obstetricians and Gynecologists* (ACOG) recommends encouraging optimal control of obesity before pregnancy [4]. However, this recommendation often goes unnoticed since more than half of American women with a live birth in 2020 were in overweight or obesity condition [3]. To make matters worse, pregnant women with overweight and obesity more frequently experienced pregnancy weight gain above the recommended range of the IOM [5].

Thus, all efforts should be made to achieve optimal body weight control before pregnancy [2], with a minimum target of 5–10% of weight loss.

Bariatric surgery, including both restrictive and malabsorptive procedures, has become more widespread [7] and is the most effective long-term option [6]. Considering the metabolic effects of bariatric surgery, most of the literature has mainly focused on the obstetric outcomes of post-surgery pregnancy [8, 21]. However, there is relatively little information on the effects of pregnancy after bariatric surgery on trajectories of weight loss, with most studies focusing on the Roux-en-Y gastric bypass (RYGB) [10] [11] and a few related to sleeve gastrectomy [22]. Yang et al., in their systematic review and meta-analysis based on data from seven retrospective cohort studies evaluating bariatric surgery and pregnancy, did not find significant differences regards to EWL after bariatric surgery (357 pregnant vs 740 non-pregnant women) and post-bariatric surgery complications (2630 pregnant vs 23,602 non-pregnant women). Similar results were found in the retrospective study by Brönnimann et al., where excess BMI loss, postsurgical, and long-term complications were similar between the pregnancy group (40 women) and non-pregnancy group (247 women) up to 5 years after RYGB [11]. Comparable 5-year results were also highlighted by Harrod et al. in their cohort of 727 women who underwent RYGB or laparoscopic adjustable gastric banding (LAGB), with 80 of them becoming pregnant after the procedure [9].

Our study is the first to investigate the relationship between ESG, a restrictive and organ-sparing endoscopic procedure, and pregnancy. We found that ESG allows for adequate weight loss (between the 5 and 10% TBWL target) before pregnancy with an excellent risk/benefit ratio, which may be helpful, especially for those women with difficulty in becoming pregnant, such as those affected by PCOS, [19, 23] as PCOS is a multifactorial condition often associated with excess weight/obesity, that appears to be associated with a reduction in fertility [24]. Moreover, in our cohort of patients among the 18 women with a reported desire for maternity associated with difficult attempts in the previous 12 months, 16.6% (3/18) managed to start and carry out a pregnancy. These results, although based on simple anamnestic investigations and not specific hormonal/gynecological investigations, could pave the way for the role of bariatric endoscopy in the multidisciplinary management of female infertility associated with overweight/obesity.

During pregnancy, 55% of pregnant women had an increase in weight according to the recommendations of the IOM. Even more important is the fact that 77% of women with obesity at the beginning of gestation complied with the recommendations, which is higher than the 24% reported in 2015 [5]. After delivery, a gradual reduction in body weight was observed, and it was maintained up to 14 months after the delivery. Due to total weight regain, one patient underwent a second endoscopic procedure after evaluation and approval by the multidisciplinary team.

Important aspects for any bariatric surgery’s success, especially for ESG, is the change in lifestyle and the impact on QoL. We evaluated this through the BAROS score and its sub-category dedicated to QoL (the *Moorehead-Ardelt quality of life questionnaire*). We observed that both parameters improved after ESG and did not undergo substantial changes during and after pregnancy.

Many women of childbearing age question whether pregnancy after ESG could compromise its medium and long-term weight loss outcomes. The data available to us, although retrospective and limited by the number of pregnant patients, show no substantial differences between women who became pregnant after the procedure and women who did not up to 24 months after ESG.

Based on our experience, therefore, we would recommend endoscopic sleeve gastroplasty to all patients of childbearing age suffering from obesity and with a desire for motherhood with a history of failure of dietary treatment. Indeed, most patients can reach a 5% TBWL within 6 months and in the case of weight regain during pregnancy, a second endoscopic treatment is possible, which at our center is offered at least 3 months after the end of breastfeeding. Unfortunately, the data available did not allow us to compare weight trajectories between patients who became pregnant before and after the 12 months suggested by ACOG [25], having only two patients who became pregnant after this time frame. So, we warmly suggest waiting at least 12 months before embarking on a possible pregnancy after ESG.

Our study has certain limitations, specifically the retrospective nature, the absence of a complete hormonal/gynecological evaluation for patients with difficulty becoming pregnant, and the limited number of pregnant women. However, this should be considered in the national context of the reduction of the birth rate. Finally, the results in terms of post-ESG fertility could be vitiated by the retrospective nature and the choice to consider only the parameter “*difficulty to get pregnant in the 12 months before the intervention*”: in fact, it is not taken for granted that a woman with difficulty becoming pregnant in the previous 12 months the surgery persists in its intent and it is possible that other factors may have affected the outcome (e.g., partner exchange, economic issues).

Nonetheless, our study has several strengths. To our knowledge, we are the first to provide information on the impact of pregnancy on the efficacy of ESG with a relatively long follow-up period. In addition, we show that ESG allows patients to reach the recommended weight loss percentage before pregnancy and that women who become pregnant after ESG appear to be more compliant with the IOM weight gain recommendation. Finally, we show that ESG may aid women affected by PCOS infertility who wish to become pregnant.

Considering the growing prevalence of minimally invasive bariatric procedures, future prospective and structured studies (with an assessment of the hypothalamic-pituitary-ovarian axis) focusing on insulin/obesity-related infertility, especially in patients affected by PCOS, will certainly be needed based on the excellent results on the effects of ESG on metabolic syndrome and diabetes shown by Abu Dayyeh et al. [13]. Moreover, to fully assess the effects of ESG on pregnancy, it is mandatory to focus on maternal (e.g., gestational diabetes, hypertension, and preeclampsia) and neonatal (e.g., fetal growth) gestational outcomes.

Finally, since ESG causes a reduction in gastric volume without altering the vitality and secretory functions of the mucosa and changes in intestinal peristalsis, it might be interesting to evaluate the effects of this procedure on nutrient absorption during pregnancy, which can often be altered after bariatric surgery.

## Conclusion

ESG produces adequate weight loss before pregnancy in patients with obesity and is potentially a useful treatment option for those women who have difficulty becoming pregnant, such as those with PCOS. Pregnancy after ESG does not adversely affect weight loss outcomes after delivery as lifestyle changes induced by ESG are maintained after pregnancy and allow for a gradual loss of weight gained during pregnancy.

## Abbreviations

ACOG: American College of Obstetricians and Gynecologists
ASMBS: American Society for Metabolic and Bariatric Surgery
BAROS: Bariatric Analysis and Reporting Outcomes questionnaire
BMI: Body mass index
BMIL: Body mass index loss
EAES: European Association for Endoscopic Surgery
ESG: Endoscopic sleeve gastroplasty
EWL: Excess weight loss
H-INS: Hyperinsulinemia
HBP: High blood pressure
IOM: Institute of Medicine
PCOS: Polycystic ovary syndrome
QoL: Quality of life
TBWL: Total body weight loss
WL: Absolute weight loss
